# Supplementary material for: IL-1β Controls Proliferation, Apoptosis, and Necroptosis Through the PI3K/AKT/Src/NF-κB Pathway in Leukaemic Lymphoblasts
Source: Biomedicines. 2025 Dec 24;14(1):41. doi: 10.3390/biomedicines14010041 (PMC12839291; doi:10.3390/biomedicines14010041)
Supplement: Supplementary file 1 [file biomedicines-14-00041-s001.zip › biomedicines-4024499-supplementary.pdf]

Table S1. Statistical differences in viable cells and the percentage of cells in early and late apoptosis after treatment with IL-1β.

	**Treatment**		***p***
	**Control**	**IL-1β**	
Viable cells (%)	66.11 ± 7.0	89.76 ± 5.0	$p < 0.001$
Early apoptosis (%)	5.0 ± 0.08	0.90 ± 0.06	$p < 0.001$
Late apoptosis (%)	0.84 ± 0.10	0.91 ± 0.33	$p < 0.05$

Table S2. Statistical differences in the percentage of cells in early apoptosis following treatment with IL-1β alone and combined with various inhibitors.

**Treatment**	**Early Apoptosis (%)**	***p***
IL-1β	0.90 ± 0.06	$p < 0.001$
IL-1β + JSH23	0.53 ± 0.12	
IL-1β	0.90 ± 0.06	$p < 0.001$
IL-1β + PP2	0.62 ± 0.10	
IL-1β	0.90 ± 0.06	$p < 0.001$
IL-1β + Wort	0.24 ± 0.05	

Table S3. Statistical differences in the percentage of cells in late apoptosis after treatment with IL-1β alone and combined with various inhibitors.

**Treatment**	**Late Apoptosis (%)**	***p***
IL-1β	0.91 ± 0.33	$p < 0.001$
IL-1β + JSH23	1.9 ± 6.16	
IL-1β	0.91 ± 0.33	$p < 0.001$
IL-1β + PP2	0.83 ± 0.09	
IL-1β	0.91 ± 0.33	$p < 0.001$
IL-1β + Wort	0.75 ± 0.07	

Table S4. Statistical differences in the percentage of cells in necrosis after treatment with IL-1β alone and in combination with various inhibitors.

**Treatment**	**Necrosis (%)**	***p***
Control	13.64 ± 2.7	$p < 0.001$
IL-1β	5.0 ± 1.9	
IL-1β	5.0 ± 1.9	$p < 0.001$
IL-1β + JSH23	30.5 ± 3.8	
IL-1β	5.0 ± 1.9	$p < 0.001$
IL-1β + PP2	10.53 ± 2.3	
IL-1β	5.0 ± 1.9	$p < 0.001$
IL-1β + Wort	12.7 ± 1.3	
